# Women's Articulations of Aging: “Learning to Be Affected” Through Experiences in Recreational Ballet

**DOI:** 10.3389/fspor.2022.795956

**Published:** 2022-02-15

**Authors:** Allison Jeffrey, Pirkko Markula, Corinne Story

**Affiliations:** Faculty of Kinesiology, Sport, and Recreation, University of Alberta, Edmonton, AB, Canada

**Keywords:** aging, dance, ballet, new materialisms, Latour

## Abstract

In this article, we draw upon the experiences of mature recreational dancers who participated in classes facilitated by a professional ballet company and catered to older adults. Moving with 11 women through a 10-week ballet course, and immersing ourselves in the empirical material, we recognized opportunities for broadening our analysis of aging dancing bodies. Inspired by a Latourian understanding of bodies and a recent new materialist turn in humanities and social sciences, we became curious about the ways that the women were being affected by their experiences in ballet. The ballet studio, the barre, muscles, sweat, and music were all discussed as influential aspects contributing to their understandings of aging and dancing. Moving beyond biomedical prescriptions and extending socio-cultural constructions, we reveal opportunities for Latourian theory to dance with us toward re-imagining what is possible for aging recreational ballet dancers. Here, we allow the women's articulations of aging in ballet to exist as unique expressions unbound by limitations. Moving with women as they learn to become more affected through dance, we are given the opportunity to think about bodies, ballet and aging differently.

## Introduction

The demographics of the world are changing with research revealing that not only is our global population increasing, it is also aging (Krekula et al., 2017). Due to increased physical and financial health, individuals aged 55+ are not only more physically mobile and capable, they are also enjoying more involvement and influence within their communities (Southcott and Joseph, 2020). Studies on aging and physical activity, particularly those engaging qualitative inquiry, reveal how varied approaches to research can illuminate nuanced meanings that this growing population of mature adults are ascribing to their movement experiences (Markula et al., 2001; Stevens-Ratchford, 2016). In this article, we discuss aging women's experiences in recreational ballet classes to reveal insights that expand upon images typically associated with aging in a Westernized context (i.e., decline and loss) (Wainwright and Turner, 2006; Nakajima, 2011; Coupland, 2013).

Importantly, what we have come to expect of the aging process is also shifting alongside examples of healthy and even thriving older individuals who are living vibrant, creative, engaged lives as they mature. Thinking of aging women in dance is particularly relevant to our discussion. While examples of exceptional professional dancers like Martha Graham and Twyla Tharpe may extend beyond what is possible for recreational dancers, these women provide inspiration and illustrate how aging does not necessarily need to be associated with a steady process of physical and cognitive decline (Nakajima, 2011). Here, we are interested in the ways that aging recreational dancers are simultaneously absorbing and releasing their limited beliefs about their moving bodies while finding new opportunities for experiences in ballet that extend beyond the confines of their bodily boundaries.

Engaging a new materialist theoretical lens, we are intrigued by the possibilities for re-turning understandings of aging and dance through the inclusion of a broader non/human materiality that lives in the ballet studio. Throughout this study, and alongside our reading of Latourian theory, we became increasingly aware of the presence and influence of the non/human actors in the ballet studio. Therefore, this article presents itself as an initial pilot study and theoretical experimentation, the beginnings of a theoretical dance that can allow us to re-imagine deeper interconnections between women, their moving bodies, and other bodies during recreational ballet.

## Literature Review: Expanding Understandings

There is a growing body of literature on aging and dance that reveals benefits related to psychological health (e.g., Keogh et al., 2009), quality of life (e.g., Houston and McGill, 2013), posture and mobility (e.g., Judge, 2003), cognitive function (e.g., Kshtriya et al., 2015) and perceived wellbeing (e.g., Ali-Haapala et al., 2020). These studies, while drawing on different theoretical frameworks and methodologies, encourage dance as an activity that can support aging populations as they move through various physiological and cognitive changes. Though we acknowledge the contribution of all research on aging in dance, for this particular article, we were inspired by findings from qualitative analyses and their potential to reveal more nuanced understandings of women's embodied experiences.

In addition to quantitative analyses of health and wellbeing markers typically associated with aging bodies and minds (i.e., balance, memory), qualitative research further reveals that participation in dance classes can assist in challenging socio-cultural ideologies and stereotypes often associated with mature moving bodies. For example, Krekula et al. (2017) asserted that the non-verbal, organizational aspects of recreational dance classes, namely choices around choreography and timing of events, could support and/or challenge meanings associated with age. Focusing on increased access to time and different styles of movement accessible only in older age, they suggested an “age power” (p. 43) that was unique to older adults. Instead of a process of decline, these researchers present aging as a possibility to experience dance during times of the day that were previously dedicated to working and family obligations, and saw power in an aging dancer's ability to express years of accumulated wisdom through considered movement patterns.

Further, research with *women* in various dance communities including social dancing (Cooper and Thomas, 2002), jazz (Alpert et al., 2009) and ballroom (Stevens-Ratchford, 2016) reveals subtle differences in gendered experiences of aging moving bodies. Coupland (2013) described how older women dancers negotiate “watchability” (p. 3), or the extent to which they deemed their bodies worthy of a sustained audience while dancing. In Coupland's analysis, dancers revealed concerns related to the “unwatchability” (p. 21) of their aging bodies. Interestingly, in addition to critiques of their bodies, these women also expressed an appreciation for a certain “truthfulness” or “wisdom” (p. 21) they could access through their movements which they attributed to the passage of time.

In a further study with belly dance (Moe, 2014), women described how their experiences in this style of dance helped them to re-define concepts typically associated with youth (e.g., sensuality). Defying narrow definitions of aging and femininity, these women were experiencing their bodies and moving toward their own embodied understandings. These articles demonstrate how qualitative inquiry on dance with aging populations can broaden understandings of women's experiences of aging. In this article, we aim to expand upon this literature by focusing on a discipline that has received less attention in aging research, the traditionally codified practice of ballet.

While there is an increasing body of literature addressing the concept of aging in a range of dance communities, there are few studies that examine ballet as a possible physical activity for an aging population. Ballet presents an interesting topic of study as a discipline that is often associated with strict adherence to codified movements and a certain body type. The stereotypical feminine ballet body is often young and highly disciplined (Markula, 2018). Therefore, a discussion on the embodiment of ballet by older dancers presents an opportunity to expand understandings. In their Bourdieusian analysis, Wainwright and Turner (2006) found aging professional ballet dancers struggling to reconcile the dissonance of embodying dance in an aging body, when compared to the stereotypical habitus of a professional dancer (i.e., young and agile). In our discussion of women's embodiment in recreational ballet, we are interested in the unique experiences of women dancers 55+ and their possible understandings of a ballet body.

As discussed, Wainwright and Turner's (2006) ethnography with mature professional ballet dancers revealed the pain associated with an awareness that the tools of their trade (their bodies) are “crumbling to bits” (p. 237). Expanding on this, Southcott and Joseph (2020), in their interpretive phenomenological analysis of the experiences of aging women dancers, for whom “ballet and contemporary dance form their core identity” (p. 1), described how aging professional dancers are finding new opportunities to share their embodied knowledge through continued engagement in dance. They emphasized the importance of showcasing the contributions of aging dancers as “performances that have a place in the arts landscape” (p. 606) and discuss the value of inclusion in the broader creative community. Studies on aging professional ballet dancers help broaden understandings on the benefits of this traditionally codified discipline for older adults, however, research on ballet and aging remains limited.

If research on professional ballet and aging is limited, there are even fewer studies with mature recreational ballet dancers. In their study, however, Houston and McGill (2013) revealed the potential for ballet to influence the physical, psychological, social, and emotional lives of recreational dancers (aged 60-82) who were diagnosed with Parkinson's. Ballet classes in this study were led by members of a professional dance company and were catered to aging dancers with little to no previous experience of ballet. Houston and McGill (2013) discussion reveals a breadth of positive outcomes from both qualitative and quantitative methods gathered over the duration of the 12-week ballet course.

To expand beyond previous work, we find inspiration in Ali-Haapala et al. (2020) study on weekly *Ballet for Seniors* classes. Similar to Houston and McGill's study, classes were taught by professional ballet dancers and catered to a population of aging recreational participants. Engaging in action research, Ali-Haapala et al. (2020) were interested in learning strategies for facilitating pleasurable experiences in ballet for mature dancers. While stereotypes around aging dancers might prompt program leaders to design less physically demanding classes for aging adults, this article revealed that dancers in the *Ballet for Seniors* classes found more enjoyment when they felt physically, emotionally, and mentally challenged. Interestingly, the “pleasurable challenges” (p. 1) these aging dancers discussed were often attributed to “the physical and cognitive challenges that are embedded in the structure of ballet” (Ali-Haapala et al., 2020, p. 532). Ballet is most often associated with pain and discipline, however, research with mature recreational dancers is revealing that there is much pleasure to be found in the ballet studio (Kolb and Kalogeropoulou, 2012; i.e., Ali-Haapala et al., 2020).

As mentioned, projects on ballet and aging have utilized qualitative, quantitative and mixed-methods approaches with each approach offering valuable insights into the complex experience of aging whilst dancing ballet (e.g., Coupland, 2013; Krekula et al., 2017; Olsson and Heikkinen, 2019; Ali-Haapala et al., 2020; Southcott and Joseph, 2020). Where we see an opportunity to expand upon previous ballet and aging research is in working with those dancers who are interested in ballet as a recreational pursuit. We are intrigued by the potential for the discipline of ballet to unveil nuanced understandings related to women's embodied experiences of dance and aging. Further, we recognize potential to expand upon the aforementioned literature, re-defining aging and recreational ballet through engaging a new materialist theoretical lens.

## Theoretical Framework: Engaging a New Materialist/Latourian Lens

Theoretically, this article draws from the new materialist turn in social sciences and humanities that investigates how the material interacts with the social in a world where both human and nonhuman are influential factors contributing to lived experience (e.g., Coole and Frost, 2010; van der Tuin and Dolphijn, 2012; Fox and Alldred, 2017; MacLure, 2017). In recent years, the body of literature engaging theories of new materialisms continues to expand understandings related to moving bodies (e.g., Markula, 2019; Thorpe et al., 2020; Fullagar and Pavlidis, 2021; Jeffrey et al., 2021; Lupton and Willis, 2021). Scholars engaging theories of new materialisms with and through moving bodies are consistently re-turning their research processes to consider natural-cultural entanglements, the agency of materiality, and an embodied experience that is continually becoming. Herein, we recognize that the materiality of dance is not separate from the embodied, felt, affective, social, cultural, environmental experiences of aging women and are interested in the ways that this complex entanglement is being felt through the discipline of ballet.

### The Socio-Materiality of Ballet

In this article, we recognize ways that research on ballet and aging has the potential to include both the material (i.e., the physical dancing body, the floor, mirrors, clothing, barre) and the social in its analysis. One of the few examples of research that draws upon new materialist theory in this context is Clark's (2020) Baradian analysis of ballet bodies. In her article, she revealed the intricate intra-actions of material and discursive forces that influence ballet dancers. As an experienced dancer, she described her process of engaging new materialist theory acknowledging the added depth and “liveliness” she was able to access through this theoretical lens. She wrote:

*Indeed, knowing and thinking about ballet bodies through Barad's onto-epistemological framework allowed me to understand ballet bodies as more than discursive constructions and accommodated the liveliness I sensed in my original dissertation study but was unable to adequately theorize (p. 221)*.

Similar to Clark, we recognize the potential for new materialist theory to broaden discussions beyond discursive construction, opening toward a curiosity around the agency of both human and nonhuman elements in ballet. Clark described a desire to move beyond critiques of representational ballet bodies influenced by structures of power. In revisiting her empirical material, she became intrigued by the “vitality of the ballet body, and the relationships it created with the other bodies, things and spaces around it” (Clark, 2020, p. 210). This led her to engage with Barad's (2003, 2007) writings as a theoretical framework that enabled her to include a discussion on the “dynamism of matter” (p. 210) and the intricately entangled elements of matter, meaning and discourse in ballet. Sensing and describing the inseparability of the material-discursive through her Baradian engagement, Clark (2020) revealed how “ballet bodies become through their movements and relationships with other bodies” (p. 221), both human and non.

The materiality of ballet and some of the complexities contained within this particular movement culture are also featured in Markula's (2021) Latourian analysis of barre classes (a hybrid ballet-fitness formation). Engaging a new materialist lens, Markula described how Latour's actor network theory (ANT) reveals the human/nonhuman elements acting within this hybrid ballet-fitness formation. Markula (2021) began with a discussion of the aesthetic, historical, social and cultural implications pressing upon moving bodies as they discipline themselves toward fitting the norms of both ballet and fitness communities. She added complexity to this discussion through her consideration of the material presence of the barre. Her Latourian analysis, as mediated by the barre, enabled Markula to re-imagine moving bodies as phenomena that are influenced by both human and nonhuman actors in hybrid ballet-fitness classes.

In our article, we are inspired by these previous new materialist re-imaginings of ballet and hybrid ballet-fitness forms, as well as current themes in new materialist research on physical cultures more broadly (i.e., Lupton, 2019; Fullagar, 2020; Thorpe et al., 2020). Where Markula (2021) and Clark (2020) located a richness in their discussions whilst writing through matter, we see an opportunity for our discussion of ballet and aging to locate a certain depth through a Latourian consideration of affect. Recent new materialist research (i.e., Fullagar and Pavlidis, 2021; Jeffrey et al., 2021) reveals how embodied and complex *affective* relations, as “capacities and entangled moments that moved us to know differently” (Fullagar et al., 2021, p. 176), can assist scholars in uncovering intricate details around movement and leisure practices that are inclusive of both human and nonhuman materiality. Scholars invested in a wide variety of research are increasingly engaging the term affect from a range of theoretical, ontological, epistemological and disciplinary approaches (Gregg et al., 2010). Current analyses of affect are not derived from a unified approach to studying emotions, feelings, and bodies. Therefore, it is important to explicitly state how we define affect in this article. In our research with aging women participating in recreational ballet, we engage a Latourian understanding of affect, not as an emotion, but as a force that enables us to consider how these women's embodied experiences were inclusive of social, cultural, and material factors being lived through their aging moving bodies. We engage Latour's (2004) description of affective learning as a theoretical lens that enables us to think about the body differently. Inspired by Latour's “body talk,” we carefully consider how affective experiences illuminate different understandings of aging bodies as social, material, cultural, non/human experiences that exist in perpetual motion.

### Latour's “Body Talk”

In his article, Latour (2004) reflected upon a conference session where he asked attendees to write down an antonym for the word “body.” Prompted by the responses, he offered a framework for talking about the body that challenges distinctions of human and nonhuman, liveliness vs. death. Extending upon previous theories, Latour (2004) described the body as, “*an interface that becomes more and more describable as it learns to be affected by more and more elements*” (p. 206, emphasis in original). Due to the complexity of his discussion and the increasing presence of the term “affect” in academic research (e.g., Braidotti, 2020; Fullagar and Pavlidis, 2021; Jeffrey et al., 2021), it is important to explicitly clarify how we define and engage the term affect in our research. Here, when we talk of affect, the focus is on what the “body has become aware of” (Latour, 2004, p. 206). The body is not theorized directly, rather, it is understood as being acquired through a process of becoming increasingly affected, “a progressive enterprise that produces at once a sensory medium and a sensitive world” (p. 207). Central to Latour's approach to talking about the body, and to our analysis of the empirical material, is what is described as a process of “learning to be affected” (Latour, 2004, p. 209). It is through Latour's description of the process of learning to be affected that we can illustrate how we understand affect in this article.

Through the example of training a nose in the perfume industry, Latour (2004) described the ways that the sensory, material, human and nonhuman all contribute to an ongoing training in affectivity that results in a more affected nose. Included in his description of this process of becoming more affected are the odor, the nose itself, brain activity, chemicals, measurement apparatus, teachers, and time. Latour revealed that through training and repeated exposure to different odors, the perfumier becomes more sensitive to subtleties, and eventually begins to “inhabit a (richly differentiated odiferous) world” (p. 207). According to Latour, learning to be affected is an ongoing process where “body parts are progressively acquired at the same time as ‘world counter-parts' are being registered in a new way” (p. 207).

Through this affective training, the increasingly expansive experience is unique to each particular nose. Latour (2004) described the individualized experiences and expressions of increasingly affected bodies as “articulations” (p. 206). Articulations arise as unique and ongoing processes of becoming. They shift alongside new information being processed during the external/internal, human/nonhuman experience of learning to be affected. Through articulations, increasingly affected bodies are able to bring voice and meaning to the conditions being experienced. Latour's understanding of articulations allows for difference through acceptance of multiplicity and controversy as integral in broadening understandings of the world. In his description of the complex and multiple layers of difference, he insisted that “the more you articulate controversies, the wider the world becomes” (Latour, 2004, p. 211). The tensions present in nuanced experiences of the world are encouraged as articulations through Latour's body talk.

Finally, and important to our discussion of aging women's experiences in recreational ballet, is Latour's description of propositions. Latour (2004) stated, “I have acquired the habit of using the word propositions to describe what is articulated” (p. 212). The proposition exists as an entity that is continually being articulated. He continued to describe his understanding of a proposition through the example of a perfumier. In the example of an odor, the affected body is experiencing and composing an understanding of odor through an ongoing process of discovery. There is no singular definition of an odor, no stagnancy, but it articulates itself differently for each nose and shifts in relation to the human/nonhuman actors in its midst. In our discussion of aging bodies experiencing recreational ballet, we consider aging to be a proposition. We are interested in the nuanced articulations of aging that are being expressed by the women as they move in ballet classes. Further, we consider how ballet might be providing women with opportunities to increase appreciation for their unique articulations of aging while learning to become more affected through embodied experiences in recreational dance.

## Methodology: Becoming Affected With and Through Theory

This study features a partnership between a professional dance company and a university-based research team. The purpose of this inquiry was to examine mature non-dancers' experiences in ballet classes designed specifically for older adults. In collaboration, the research team and dance company advertised ballet classes catered to older adults (55+) interested in participating in research on recreational dance and aging. Classes were taught in a professional ballet studio by a company member and continued over a 10-week semester with one 90 minutes session per week in the Fall 2019. The professional dance company generously provided the ballet instructor and studio access. After receiving confirmation of ethics approval for our project by the university's research ethics board, we began recruitment. We recruited the participants with the support and assistance of the ballet company through a direct email from the ballet company, an advertisement on the company's social media, and/or through word of mouth. In recruitment advertisements, the ballet class was described as catered to individuals aged 55+ and did not require previous ballet experience. Participants consisted of 11 (12, with researcher) older (55+) women with varying expertise in ballet ranging from spectators to experienced dancers. In this paper, we assigned pseudonyms for each of the women and used these consistently throughout.

### Methods

In this study, we used qualitative methods to broaden our understandings of aging women's experiences of recreational ballet. Due to our desire to attain rich empirical material, we concluded that interviews and participant-observation were the most appropriate methods for attaining in-depth insights on women's embodied experiences in recreational ballet (e.g., Patton, 2002; Markula and Silk, 2011; Brinkmann and Kvale, 2015). In this article, however, we draw upon the empirical material from the interviews.

Semi-structured interviews were conducted to obtain in-depth information regarding participants', as well as teachers', experiences of the ballet class (e.g., Patton, 2002; Markula and Silk, 2011; Brinkmann and Kvale, 2015). However, for the purposes of this paper, we focus solely on the participants' experiences. At the end of the 10-week semester of classes, all of the women were invited to participate in interviews. The interview guide was informed by previous literature, as well as experience and insights of the researchers drawing upon class observations. Individual face-to-face interviews, with durations ranging from 30 to 60 minutes, were conducted in a participant's home or in a cafe chosen by the participant.

The interviews were transcribed verbatim for analysis (e.g., Patton, 2002; Markula and Silk, 2011; Brinkmann and Kvale, 2015). We analyzed the empirical material through a five-step process informed by our new materialist theoretical approach (Markula and Silk, 2011). This resulted in the following main themes: aging as a proposition, articulations of aging in ballet and learning to be affected.

## Findings

Through our discussion, we illustrate the women's nuanced experiences in ballet and highlight the human and nonhuman aspects contributing to their understandings of both themselves, of their bodies, and of the material presence of other actors in the ballet class through an engagement with Latour's (2004) body talk. Our findings are distributed into three sections inspired by different Latourian concepts that are central to how he re-imagined discussions of bodies. The first section considers aging as a *proposition*, a concept without a singular definition that is taking shape and shifting alongside socio-material factors influencing women's individualized experiences of it. The second considers the women's unique *articulations* of aging moving bodies as experienced in the context of ballet. Finally, we reveal how recreational ballet classes are supporting aging women in *learning to be affected*, as processes of moving with and simultaneously being “put into motion by other entities, humans or non-humans” (Latour, 2004, p. 205).

### Aging as a Proposition

For Latour (2004), a proposition is a continually becoming phenomenon that is articulated through ongoing inquiry. Following Latour's description of the crucial elements required for a consideration of a proposition, we illustrate how aging can be a position that has no definite distinction, yet in its multiplicity retains some solidity. Here, we re-imagine how aging as a proposition, and as felt through recreational ballet, became increasingly complex as the women navigated the natural/cultural phenomena of their aging moving bodies.

When discussing age, many participants mirrored sentiments that resembled stereotypical discourse around aging as a process defined by loss and decline (Stevens-Ratchford, 2016; Krekula et al., 2017). When asked about the benefits they saw in maintaining movement through aging, Jan understood that “it's something we need to be doing at our age.” Similar statements around the necessity of instilling habits that could counter the physiological changes that occur with age were mentioned by many of the women. Below, Sandy attributed her morning movement routines to reducing the effects of aging:

*I start moving before I even get out of bed, I do my own little bed exercises, you know something that I can do so I can get up and I don't hurt and I can actually walk without pain. So, I think that I see other people can't walk and it just keeps me, I don't want to say younger, but maybe it keeps me from aging as quickly as say someone who's not*.

Sandy's desire to counteract the perceived negative influence of the aging process and maintain some semblance of youth through her movement practices illustrates how she associates aging with reduced mobility and increased pain. In addition to concerns around physical decline, there was also an awareness of the potential for the aging process to potentially infringe on personal freedoms and general autonomy. As Nancy reflects on the potential health benefits of ballet, she states:

*I think the health benefit would just be contributing to a sense of having control. That you can do things, and get better, and so on*.

Nancy, like many of the women, was concerned with maintaining autonomy in older adulthood and considered continued movement to be integral in her ability to live well on her own terms. According to Nakajima (2011), cultural context greatly influences perceptions of age and impacts ideologies around what is considered possible in aging moving bodies. In Japan, the aging body is often viewed as masterful. In the culture of North America, neoliberal discourse influences discussions on aging with ideological assumptions that age necessarily includes restricted mobility and a general decrease in physical capability (Coupland, 2013). The women mirrored these assumptions in their discussions around losing control and counteracting this with movement practices and mobility maintenance. Many of the women in our study were concerned with the threat of physical decline and this revealed itself through their regular mention of balance and falling:

*Sandy: I know of a wife of a friend of my husband's. She's a bit older than me […] she used to be very active but she's so heavy now and if she falls, she can't get up. And nobody wants to be in that position. I never want to be in that position. I'd rather die before that happens*.

*Linda: I know that working on the balance and the flexibility and the endurance is critical […] and I realize the risk of falling, as I've fallen a couple of times. I mean I feel ancient. I feel ancient saying that because I worked in the health system for years, and I remember seeing those posters about falling, fall prevention […] and before you know it, you're there*.

The loss of balance, flexibility, or a changing body shape emerged as a serious threat to these women's quality of life. Sandy was even prepared to die if she could no longer be mobile and thus, “working on” mobility was the key, particularly when feeling “ancient.” In addition to concerns related to physiological changes, negative changes to cognitive and social aspects of their lives (e.g., dementia, isolation) were also discussed. Jean describes how attending ballet classes, “is important as you get older because people can kind of almost shut themselves away.” Sandy reflects on the experience of watching her mother age and considers dance to be a potentially remedial activity, “I don't want to have the dementia that she has so maybe dancing can ward that kind of thing off.”

Importantly, and in addition to these concerns, the women also had many realizations about their moving bodies that expanded beyond ideological assumptions of aging and worries about imminent decline. Through their involvement in recreational ballet classes, these women were given the opportunity to access enjoyment and this pleasure was inclusive of human and nonhuman elements. Aging, as a proposition, was shifting alongside these women's experiences in ballet. In feeling their muscles and bodies moving in new ways, and through immersing themselves in spaces for dance outside of societal expectations related to age, these women (with little to no prior experience in ballet) allowed their propositions of aging to shift beyond their concerns and toward new opportunities for joy. This is illustrated in Michelle's reflection below:

*I actually really liked using the barre. Maybe, it's tied to the balance thing, but I really enjoyed stretching with that security. I enjoyed the music. I enjoyed the sense of movement, I suppose learning a little bit of ballet […] the instructor, the classroom itself, the physicality of it, the participants. Very welcoming group of ladies, all different sizes, body types, backgrounds, but all common in the sense that we were the same age and we weren't in amongst a bunch of young girls and men who were checking themselves out in their lululemon outfits […] it felt comfortable being amongst everybody*.

Michelle mentions a wide range of influences (both human and nonhuman) that were all contributing to her enjoyment of ballet. Moving beyond Ali-Haapala et al.'s (2020) discussion of experiencing pleasure through challenging ballet choreography, these women were finding enjoyment in the environment, the connections with other women, the music and the movements. As recreational ballet dancers, they were not confined to specific ballet clothing or any other codified limitations often associated with ballet and this greatly influenced their ability to find joy in their entire socio-material experience. Aging, as a proposition, was being enjoyed as they moved in the ballet studio with other mature recreational dancers. Outside of limiting discourse around how aging *should* be expressed and experienced, these women revealed an enjoyment of moving in the ballet studio and were influenced by their entire human/nonhuman experience. Below Nancy and Jill describe aspects of ballet classes that influenced their enjoyment:

*Nancy: I mean my favorite, in the sense of doing things to music, was on the floor. It was more gentle and enjoyable. But another favorite was the barre, some of the leg lifts and the things that really got at those new muscles that I don't often use. I didn't like it, but in a way, it felt really good*.

*Jill: I enjoyed the class and found that it went quickly. She [the instructor] really did a great job of kind of changing it up. You know, interspersing the weights with the barre. I thought it was really well timed and well paced for our group too, moderate intensity, lower intensity*.

These women's experiences of aging in ballet broaden to include experiences of entangling with materiality as joyous and this extends beyond concerns of aging and decline. Nancy enjoyed the barre and Jill the exercise with the weights, both material objects that added special elements to their movement practice. Latour (2004) described how these processes of creating propositions (e.g., aging) exist beyond expectation. He further suggested how broadening our views beyond scientific measurements of moving bodies (e.g., physiological measures of decline), can allow for a more complex “multiverse,” as a “*universe freed from its premature unification*” (Latour, 2004, p. 213, emphasis in original). Moving in a universe that is multiplicitous, we were more able to illustrate the women's *articulations* of ballet as extremely nuanced embodied experiences.

### Articulations of Aging in Ballet

Articulations, in a Latourian sense, reveal unique embodied experiences of aging that are continually in motion, responding to environment, classmates, personal, and societal expectations, as well as physical, mental, and material factors present in the ballet studio. Previous dance literature with both younger and older dancers discusses embodied experiences as complex entanglements that are influenced by many factors including social, physical, mental, spiritual and material elements (i.e., Houston and McGill, 2013; Barbour et al., 2020). Expanding upon this previous literature, we engage Latour's concept of articulation to reveal how propositions of aging are being expressed through moving bodies as continually becoming forms that are simultaneously social, physical, mental and material. Jean describes how her experiences in ballet are simultaneously physically and mentally stimulating:

*In doing those exercises, it helps me to focus and to try to remember the sequence of things that I've done. I have a real issue, problem with numbers for instance, it's just a scramble, and it doesn't matter whether it's a phone number or looking at the price of something […] the overall effect of exercise, I think, for me it's a really big deal*.

Jean, a woman concerned with cognitive decline, discussed the benefits of a mind-body connection that she experienced during dance. Janice and Linda expand upon this to describe the ways that recreational ballet classes have influenced their confidence and self-awareness:

*Janice: It [ballet] makes me feel more confident, it makes me feel like I can tackle more things. It makes me feel less like I'm aging. It makes me feel engaged and connected*.

*Linda: I see that it [ballet] can give you posture and more grace in your movement and stability and just generally improves your sense of self*.

The women's descriptions above allude to connections and benefits that extend beyond biomedical markers for *success* in aging. These broader understandings of health and wellbeing are not new and have been discussed in literature on aging and dance (e.g., Andersson and Almqvist, 2020; Southcott and Joseph, 2020). Drawing upon Latour's (2004) recommendations for allowing increasingly complex articulations to reveal wider worlds, we add a discussion of the inanimate materiality that was also influencing women's experiences of recreational ballet.

Recent research on moving bodies are considering the ways that our movement, wellbeing and health may be deeply interconnected with our environments (Barbour et al., 2019), human/nonhuman materiality (Thorpe et al., 2020), socio-cultural discourse (Markula, 2014), and affective experiences (Jeffrey et al., 2021). Some of the women in this study revealed an increased awareness of their bodies as being affected by a range of human/nonhuman actors. Below, Nancy and Sarah discuss challenges in class and explain the varied ways that the movements and the ballet studio itself were all influencing their aging bodies:

*Nancy: I was challenged with balance for sure, and I think, if you can call it endurance. Just sometimes just getting to the end of the count and the music. I mean, I don't sweat much, but in that class I did. And there were after effects, there were certain muscles that you could really feel the next day, but not inappropriately. Just, I could tell that I pushed more than I would usually push myself*.

*Sarah: I think anything to do with the balance, being on one foot. I stayed close to the wall because of that, just so I could touch something*.

These women described being affected by more than just the movements and the other participants, they regularly included mention of the music, the studio floor, the wall, the barre, and discussed the influence of these elements on their embodied experiences. Both found the class physically challenging, but appropriately so. For Sarah, it was possible to perform the balance exercise with the support of wall whereas Nancy found herself “pushing” herself beyond her usual commitment to exercise. Similar to Clark's (2020) process toward recognizing layers of inquiry beyond representational analyses of balletic bodies, we too see opportunities to expand and consider women's embodied experiences beyond physical, social and cultural constructions. We became curious and began paying close attention to the ways in which they were languaging their answers, considering how the descriptions of their complex articulations of aging in ballet were inclusive of human and nonhuman elements.

As we moved deeper into the analysis of our empirical material, we noticed moments when the women turned to metaphor to describe intricacies in their experiences. Unique to the experiences of this group of women, were the ways in which they articulated the feeling of aging as one of tightening, which the ballet class started to loosen. Lina recalled, “feeling so much less knotted up” after class and many of the other women expressed how movements incorporating flexibility were most appreciated. In the example below, Sandy compares her felt experience of aging in her moving body to seasonal changes she experienced in nature:

*I feel like you know all of my joints and all of my muscle fibers are atrophying and it doesn't feel good. And when I do finally get back into moving, it's sort of like the long winter, especially when you get into the end of March […] you just feel like everything's kind of congealing, and as soon as you get out into the space, and start moving, your body goes “yeah let's do this more.”*

Sandy's poetic description compares experiences in ballet with a liveliness that is experienced in nature. She now proposes the opportunity to re-define the purpose of movement as we age. Imagining aging bodies to be experiencing winter, then re-imagining them accessing a sense of the vitality of spring through ballet offers a metaphor that helps to accentuate the complexity of the women's experiences and the potential for dance to support healthy aging beyond biomedical and remedial aims.

Using poetic language and including a discussion of affect allowed the women, and ourselves, to access a wider range of human/nonhuman elements all contributing to the expanse of articulations being uniquely expressed through women's experiences of aging and dancing ballet. This consideration of language is important in research that engages new materialist theory. We expand on this below as we discuss Latour's (2004) process of becoming more affected and reveal the women's experiences of learning to be increasingly affected through experiences in ballet.

### Learning to Be Affected

Through this study with recreational ballet dancers, we recognized how the women's embodied experiences were facilitating opportunities for them to become more affected by the range of human/nonhuman, unique/shared processes of aging. Therefore, in this final findings section, we consider how recreational ballet served as a training in becoming more affected, a re-learning that brought women into contact with an increased sensitivity and awareness of the agency of both human and nonhuman matter in the ballet studio.

As indicated by Barbour (2011) and Humberstone (2013), there is a tension that arises when situating extremely nuanced embodied experiences into the confines of language and the structures of academia. In addition, and as discussed above, there are various applications and interpretations of terms, like affect, that assist researchers in grappling with languaging human/nonhuman entanglements (i.e., Braidotti, 2020; Fullagar and Pavlidis, 2021; Jeffrey et al., 2021). Inspired by Latour (2004), we understand bodies to be processes that exist in continual motion, sensory mediums that are simultaneously producing sensitive worlds. Latour's “learning to be affected” (p. 209), thus, is a process whereby the body is continually being re-constructed, put into motion through an ongoing relational experience that is inclusive of both human and nonhuman actors. Many of the women mentioned how their experiences in dance increased their sensitivity. Below, Sandy recalls how ballet was influencing her felt experiences in her body:

*Moving always makes me feel better. You know? Maybe I'm down. Or maybe I'm feeling like I'm catching a cold or something. If I just start moving and sweating and getting involved in how my body feels, when I move, it always makes me feel better*.

Becoming more aware of her felt experiences through class, Sandy, like many of the other women, discussed an increased affectivity she was accessing through ballet. Sandy, as one of the women with slightly more experience in dance, further expressed how these ballet classes were facilitating a bodily “awakening” for this group of older women:

*I think sort of awakening and utilizing muscles that aren't always used. Certainly for the average woman over 55 […] I think that what was done in this class really helped to strengthen different muscle groups, that maybe some of these ladies haven't been in touch with for a while, and that's a good thing*.

Sandy expressed how these classes were challenging the women and helping them to become more aware of their bodies through challenging different muscle groups. Furthermore, exploring different aspects of their felt experiences in ballet, many of the women subtly alluded to an increased ability to monitor their movement practices to suit their needs. Linda recalls an awareness of shifting daily experiences in her body and in movement:

*As I said, there were some things I just couldn't do, and the balance pieces were really challenging for me, which shows me how much work I need to do […] but generally, and of course like anything else, some days you're better than others. You know? Some days you're more rested, or whatever, and you just know*.

Linda's comment reveals the ways in which she continually reflected upon and sensed the capabilities of her body in each moment and movement. The teacher of this ballet class encouraged the women to adapt and adjust the movements to suit their needs and this may have enabled the women to further pay attention to their bodily sensations. Paying close attention to their embodied experiences, the women were learning to be more affected and increasing their awareness of their physical and cognitive capacities in ballet. In addition to paying attention to their bodily sensations, becoming more affected included a consideration of sensory elements and inanimate materiality also situated in the ballet studio. When Janice was asked to describe her favorite aspects of class, she did not discuss her individualized emotions and/or physical sensations, rather she took the opportunity to describe the influence of the studio:

*I thought the space was beautiful. It was well lit, it was clean […] it wasn't dusty or musty. It was a beautiful space. That made me comfortable. Good lighting and nice music*.

Janice described the influence of the atmosphere on her embodied experience and the ease she was able to access through the aesthetics of the studio space and the music. The ambience of the space greatly influenced many of the women. Below, Jean, like Janice, recalls the influence of the music on her experience of class:

*For me classical music, it's calming […] and if I can close my eyes then it sort of takes you to a whole other place, whatever your happy place is, you know?*

For Jean, the music took on a transcendent quality, influencing her emotions and transporting her to a “happy place.” The process of learning to be affected can prompt enjoyable experiences, as discussed, but it can also incite those that are less enjoyable. For these women, the process of becoming more affected was inclusive of inanimate materiality, sensuous moments of pleasure, and those of discomfort. Ballet for these women was equally enjoyed and resisted as the women pressed against the limitations they experienced in their moving bodies. When faced with the mirror, both Joan and Linda expressed conflicting sentiments:

*Joan: If there were no mirrors, I would love that. Just because I don't like my body image right now. But I think it's important because you can see your form*.

*Linda: It's always trouble to have to confront your body, but helpful, because it does allow you to see your posture, how you're standing or how you're lifting your arms*.

Whether these women's sentiments were indicative of their concerns with their aging bodies, or whether these bodily judgements have persisted throughout their lives, is unclear. Through Joan and Linda's comments, we recognize that these women are influenced by the feminine body ideal (i.e., young and thin). Joan does not like her body image and Linda has trouble confronting her body in the mirror. While these women's concerns are not different from previous discussions on mirrors in mature dance classes (e.g., Coupland, 2013), they illustrate that not all experiences in human/nonhuman movement practices are successful, beneficial or even enjoyable.

Moments of non-enjoyment and tension whilst dancing ballet reveal how the process of becoming more affected can be experienced both positively and negatively. The women in this study mentioned enjoying ballet and expressed how these experiences enabled them to access parts of themselves that they had not felt in years. Their experiences were not always enjoyable, but they always provided the opportunity to become moved or increasingly affected by their experiences. To finish our discussion, we offer a reflection from one of the authors after a session she attended. Below, she describes the stark differences felt by the women as they danced in different studio spaces:

*When I walked into the building, there were seven participants visiting with each other in the common area on the main floor. Our studio was in the basement, it had green walls, and no natural light or windows of any sort. I asked them, “are we in the upstairs studio today?” They replied, “no, we just don't want to go down into the dark yet.” Two weeks later, due to construction in the building and an overpowering smell of gas downstairs, our class moved into a studio upstairs. The participants jokingly said they were “graduating to the next level.” Moving upstairs into a beautiful studio with a real piano and many windows for natural lighting and artwork, impacted us. Everyone was delighted*.

In this reflection, there is an acknowledgment of the light, the windows, the space, art, color of the walls, thus highlighting the range of elements that were all contributing to the women's processes of becoming more affected through ballet. Through an increased awareness of the complexity of any experience of aging and dance as not only a physical, mental, cultural phenomenon, but also as a material, discursive, natural, felt experience assists us in questioning the limitations of previous scholarship. Through this initial pilot study, we revealed how experiencing ballet in mature bodies can revitalize sensory mediums attuning them to their surroundings and the influence of all matter in the studio, not solely those aspects traditionally associated with ballet. These findings spark new curiosities that we bring with us into the ballet studio as we expand beyond this initial pilot study.

## Conclusion

Through our Latourian analysis of aging women's experiences in recreational ballet, we revealed new possibilities for mature populations in dance classes. Thinking beyond aging as a process of decline, this inquiry led us to recognize the ways that ballet was providing these women with opportunities for becoming more affected. The articulations they revealed through their expressions of aging were unique and challenge us to consider how women's experiences of aging in the context of ballet cannot be neatly defined in monolithic terms. Aging in ballet can be expressed as a process that is simultaneously experienced as decline and expansion, wisdom and defeat. It can be all and none of these articulations at any one moment. As this article suggests, women's experiences of aging and ballet are complex, embodying multiple articulations, and continually becoming. Learning to be increasingly affected, the women are allowing the proposition of aging itself to exist as an intangible, expansive, complex phenomenon. These findings have particular implications for the study and practice of recreational ballet. This initial study provides inspiration for our continued research on women's experiences of dance and aging. We recognize great potential in designing classes that consider the broader socio-materiality that can be accessed in the studio space both for dancers and researchers of dance.

We acknowledge that we cannot contain the breadth of women's experiences of aging and recreational ballet in one article, nor can we attempt to bring the expanse of Latourian theory into the confines of tidy sentences. However, what this study aims to initiate, is the beginning of a curiosity. The possibilities contained in a Latourian, and even more broadly in a new materialist, theoretical framework are limitless. Engaging a new materialist framework from initial design through to analysis in our future research projects, we aim to access more in-depth discussion on the range of non-human materiality influencing women dancers. Through careful consideration of studio space, clothing, music, and movements, we anticipate more expansive experiences for women dancers and more comprehensive analyses of the ways in which the socio-materiality of dance influences women's experiences of aging. These initial intrigues are discussed with the hope of sparking continued discussion around experiences of aging and the possibilities for older recreational dancers in ballet. The theoretical underpinnings of our thought processes guide both our individual movement practices and our lives as researchers. As such, this article serves as a starting point in our philosophical and practical engagements. In our future studies on women's experiences of aging in dance, we imagine designing projects that allow deeper insights as we continue to age, move and question the vastly interconnected world we are dancing within.

## Data Availability Statement

The datasets presented in this article are not readily available because all empirical material remains confidential and is stored in accordance with University of Alberta ethics protocols. Requests to access the datasets should be directed to adjeffre@ualberta.ca.

## Ethics Statement

The studies involving human participants were reviewed and approved by University of Alberta Research Ethics Board. The patients/participants provided their written informed consent to participate in this study.

## Author Contributions

AJ, PM, and CS collaborated on this article. PM designed the study with CS participating as an RA in the project. AJ completed a secondary analysis of empirical material and led the writing of this manuscript. PM contributed to the overall crafting of the document, providing feedback and suggestions throughout. All authors contributed to the article and approved all edits.

## Funding

This research was provided by the University of Alberta EFF-SAS funding. The publication is funded by Social Sciences and Humanities Research Council Canada Connection Grant #611-2019-0314.

## Conflict of Interest

The authors declare that the research was conducted in the absence of any commercial or financial relationships that could be construed as a potential conflict of interest.

## Publisher's Note

All claims expressed in this article are solely those of the authors and do not necessarily represent those of their affiliated organizations, or those of the publisher, the editors and the reviewers. Any product that may be evaluated in this article, or claim that may be made by its manufacturer, is not guaranteed or endorsed by the publisher.
